# Outbreak of *Burkholderia multivorans* among patients at two acute-care hospitals in California, August 2021–July 2022

**DOI:** 10.1017/ash.2023.353

**Published:** 2023-09-29

**Authors:** Kiara McNamara, W. Wyatt Wilson, Dipesh Solanky, Sophie Jones, Elizabeth Ohlsen, J.B. Bertumen, Mark Beatty, Heather Moulton-Meissner, Paige Gable, John LiPuma, Grace Kang, Margaret Turner, Erin Epson, Kiran Perkin, Raymond Chinn, Jane Siegel

## Abstract

**Background:** *Burkholderia multivorans* are gram-negative bacteria typically found in water and soil. *B. multivorans* outbreaks among patients without cystic fibrosis have been associated with exposure to contaminated medical devices or nonsterile aqueous products. Acquisition can also occur from exposure to environmental reservoirs like sinks or other hospital water sources. We describe an outbreak of *B. multivorans* among hospitalized patients without cystic fibrosis at 2 hospitals within the same healthcare system in California (hospitals A and B) between August 2021 and July 2022. **Methods:** We defined confirmed case patients as patients without cystic fibrosis hospitalized at hospital A or hospital B between January 2020 to July 2022 with *B. multivorans* isolated from any body site matching the outbreak strain. We reviewed medical records to describe case patients and to identify common exposures. We evaluated infection control practices and interviewed staff to detect exposures to nonsterile water. Select samples from water, ice, drains, and sink splash zone surfaces were collected and cultured for *B. multivorans* in March 2022 and July 2022 from both hospitals. Common aqueous products used among case patients were tested for *B. multivorans*. Genetic relatedness between clinical and environmental samples was determined using random amplified polymorphic DNA (RAPD) and repetitive extragenic palindromic polymerase chain reaction (Rep-PCR). **Results:** We identified 23 confirmed case patients; 20 (87%) of these were identified at an intensive care unit (ICU) in hospital A. *B. multivorans* was isolated from respiratory sources in 18 cases (78%). We observed medication preparation items, gloves, and patient care items stored within sink splash zones in ICU medication preparation rooms and patient rooms. Nonsterile water and ice were used for bed baths, swallow evaluations, and ice packs. *B. multivorans* was cultured from ice and water dispensed from an 11-year-old ice machine in the ICU at hospital A in March 2022 but no other water sources. Additional testing in July 2022 yielded *B. multivorans* from ice and a drain pan from a new ice machine in the same ICU location at hospital A. All products were negative. Clinical and environmental isolates were the same strain by RAPD and Rep-PCR. **Conclusions:** The use of nonsterile water and ice from a contaminated ice machine contributed to this outbreak. Water-related fixtures can serve as reservoirs for *Burkholderia*, posing infection risk to hospitalized and immunocompromised patients. During outbreaks of water-related organisms, such as *B. multivorans* , nonsterile water and ice use should be investigated as potential sources of transmission and other options should be considered, especially for critically ill patients.

**Disclosures:** None

